# Vaccinia Virus Protein B18R: Influence on mRNA Immunogenicity and Translation upon Non-Viral Delivery in Different Ocular Cell Types

**DOI:** 10.3390/pharmaceutics13010074

**Published:** 2021-01-07

**Authors:** An-Katrien Minnaert, Joke Devoldere, Karen Peynshaert, Laure Vercruysse, Stefaan C. De Smedt, Katrien Remaut

**Affiliations:** 1 Lab of General Biochemistry and Physical Pharmacy, Faculty of Pharmaceutical Sciences, Ghent University, Ottergemsesteenweg 460, 9000 Ghent, Belgium; ankatrienpaula.minnaert@ugent.be (A.-K.M.); joke.devoldere@ugent.be (J.D.); karen.peynshaert@ugent.be (K.P.); laure.vercruysse@ugent.be (L.V.); stefaan.desmedt@ugent.be (S.C.D.S.); 2 Ghent Research Group on Nanomedicines, Laboratory of General Biochemistry and Physical Pharmacy, Department of Pharmaceutics, Faculty of Pharmaceutical Sciences, Ghent University, Ottergemsesteenweg 460, 9000 Ghent, Belgium

**Keywords:** mRNA, retina, retinal drug delivery, innate immune system inhibition, B18R, retinal pigment epithelial cells, Müller cells

## Abstract

In the last few years, interest has grown in the use of nucleic acids as an ocular therapy for retinal genetic diseases. Recently, our research group has demonstrated that mRNA delivery could result in effective protein expression in ocular cells following subretinal injection. Yet, although mRNA therapy comes with many advantages, its immunogenicity resulting in hampered mRNA translation delays development to the clinic. Therefore, several research groups investigate possible strategies to reduce this innate immunity. In this study, we focus on B18R, an immune inhibitor to suppress the mRNA-induced innate immune responses in two ocular cell types. We made use of retinal pigment epithelial (RPE) cells and Müller cells both as immortalized cell lines and primary bovine cells. When cells were co-incubated with both B18R and mRNA-MessengerMAX lipoplexes we observed an increase in transfection efficiency accompanied by a decrease in interferon-β production, except for the Müller cells. Moreover, uptake efficiency and cell viability were not hampered. Taken together, we showed that the effect of B18R is cell type-dependent but remains a possible strategy to improve mRNA translation in RPE cells.

## 1. Introduction

The fact that we can visualize our surroundings is a fantastic gift by nature. However, as simple as it may seem, eyesight is a complex process comprising numerous pathways and components. One of the key players is the retina, a nerve layer lining the back of the eye and composed of a diversity of cell types such as Müller cells, photoreceptors (rods and cones) and retinal pigment epithelial (RPE) cells. When light photons are absorbed by the photoreceptors, this information is transformed into an electrical signal and by the neuronal cells of the retina transferred to the brain via the optic nerve. Ultimately, this continuous communication between retina and brain provides us with the ability to see [1,2,3]. However, the function of the retinal cells can be hampered by an anomaly in one or more specific genes leading to impaired vision. This can manifest itself in a variety of ocular diseases such as Leber Congenital Amaurosis (LCA) and Retinitis Pigmentosa [4,5]. Fortunately, the accessibility and relative immune-privileged status of the eye, make it a convenient organ for gene therapy [1,5,6].

The onset of nucleic acid gene therapy already took place in 1990, when Wolff et al. showed that both plasmid DNA (pDNA) and messenger RNA (mRNA) are appropriate candidates for gene replacement or gene supplementation to treat genetic diseases [7,8,9]. Initially, pDNA attracted a lot of attention due to its higher stability but later on, mRNA was recognized as an interesting competitor due to its appealing advantages compared to pDNA. First of all, since mRNA is translated in the cytoplasm, it does not need to cross the nuclear envelope for efficient transfection. This is especially an advantage for retinal therapy since the retina harbors many non-mitotic cell types. Second, mRNA does not integrate into the genome of the host cell, creating a lower risk of insertional mutagenesis. Finally, mRNA production is straightforward and can be efficiently standardized according to good manufacturing process conditions [7,8,9,10]. As naked mRNA is rapidly degraded and does not easily enter cells, it needs a carrier system to optimize delivery. Currently, different clinical trials are studying the potential of gene replacement therapy for retinal diseases of which the majority is based on viral vectors. However, while viral vectors excel in transfection efficiency, non-viral vectors have many merits which render them interesting competitors for gene delivery. Indeed, their large carrier capacity, a low immunogenicity profile and the potential to easily adapt their molecular structure make them an attractive alternative to their viral counterpart [6,11,12]. Non-viral vectors can be divided into different classes, such as polymer-based and lipid-based carriers, describing the origin of their constituents [12]. Both Devoldere et al. and Patel et al. recently explored the potential of lipid based non-viral vectors for mRNA delivery to retinal cells and showed notable protein expression in different ocular cell types after subretinal injection in mice [12,13].

Although a lot of focus has been dedicated to the development of appropriate carriers for mRNA delivery, immune system activation remains an important hurdle to overcome [7,10,14]. It has been described before that mRNA recognition can initiate downstream signaling cascades eventually resulting in a diminished effect of the mRNA. mRNA recognition is established by Pattern Recognition Receptors (PRRs). In the endosomal compartment, Toll-like receptors (TLRs) are involved in mRNA recognition where TLR3 is assumed to recognize double-stranded RNA (dsRNA), while TLR7 and TLR8 mainly bind single-stranded RNA (ssRNA). In the cytosol, binding of mRNA is mediated by other PRRs, such as RIG-I-like receptors (RLRs). Recognition of mRNA results in the formation of transcription factors, which can in their turn initiate the production of pro-inflammatory cytokines and type I interferons (IFNs). These substances induce a new signaling pathway, eventually resulting in mRNA degradation, destabilization, and diminished translation [7,10,14].

In order to avoid initiation of the innate immune reaction, several approaches have been studied. First of all, mRNA itself can be modified by the insertion of naturally occurring modified nucleosides—such as 5-methylcytidine or 5-methyluridine—and/or several capping strategies. Changes in the mRNA backbone structure can help to avoid recognition by the several receptors and successive initiation of the signaling cascades. It has been shown before, however, that the effect of modified mRNA is cell type-dependent and can also result in a decrease in mRNA translation [7,10,14]. An alternative strategy is therefore to simulate the viral evasion of the immune system, by using small molecules to inhibit or avoid immune-related proteins. Roughly, they have been divided into two main groups, namely inhibitors that prevent production of the type I IFNs and inhibitors that suppress the effect induced by type I IFNs. Over the last years, several of these immune system inhibitors have been tested, however with variable results. [7,10,14,15].

In this research, we aim to evaluate the effect of the vaccinia-virus encoded protein B18R which acts as a decoy receptor for type I IFNs. B18R has been investigated before to improve cell viability after mRNA mediated reprogramming [10,16,17]. We wanted to evaluate if B18R can have a positive effect on the mRNA transfection efficiency in ocular cell types. To this end, we opted for two different cell types, i.e., Müller cells and RPE cells, which are encountered by mRNA complexes after intravitreal and subretinal injection, respectively [13]. In order to establish the relation between immortalized cell lines and primary cell cultures we also tested the influence of B18R in freshly isolated bovine RPE and Müller cells. To conclude, we evaluated the effect of B18R on the transfection efficiency of chemically modified mRNA. Since we used enhanced Green Fluorescent Protein (eGFP) mRNA as cargo, transfection efficiency was evaluated via flow cytometry and compared with the results of an ELISA assay, measuring the production of INF-β. Furthermore, we evaluated the influence on uptake efficiency and toxicity of the treatment in each of the different cell-types. In general, we observed a clear effect of B18R on mRNA translation in the RPE cells which could be directly linked to a decrease in IFN-β concentration. On the contrary, immortalized Müller cells did not show an increase in eGFP expression, nor a production of type 1 IFNs upon mRNA delivery. Finally, similarity between immortalized and primary RPE cells could be observed in contrast to the Müller cells.

## 2. Materials and Methods

### 2.1. mRNA and Cy^®^5 Labeling

Unmodified eGFP mRNA (Accession number: L29345) was purchased from Trilink BioTechnologies (San Diego, CA, USA). For uptake experiments, the mRNA was fluorescently labeled with Cy^®^5 using the MR3700 Label IT^®^ Nucleic Acid labelling kit (Mirus Bio, Madison, WI, USA). Briefly, the Cy^®^5 label was added to the mRNA in a 1:1 (*v:w*) ratio. Next, the reaction mixture was incubated for 1 h at 37 °C followed by purification with G50 Microspin Purification Columns (Mirus Bio, Madison, WI, USA) according to the manual.

### 2.2. Cell Culture

RPE cells were purchased from ATCC (ARPE-19, ATCC^®^ CRL-2302^TM^, Manassas, VA, USA) and cultured in Dulbecco’s modified Eagle’s medium (DMEM) supplemented with 1% 2 mM l-glutamin, 2% penicillin/streptomycin (1000 IU penicillin and 1000 µg streptomycin) and 10% Fetal Bovine Serum (FBS) (Hyclone^®^, Cramilton, UK). Cells were cultured in an incubator at 37 °C in a humidified atmosphere with 5% CO_2_. When the cells reached 90% confluency they were detached from the culture flask with 0.25% trypsin. Three days before transfection, 6 × 10^3^ cells per well were seeded in 96 well-plates.

In order to culture primary RPE (pRPE) cells, bovine eyes were obtained from the local slaughterhouse and transported in 4 °C CO_2_ independent medium (18045070, Thermo Fisher^®^ Scientific, Merelbeke, Belgium). Experimental procedure for isolation of primary RPE cells was based on the protocol of Toops et al. [18]. Eyes were cleaned by removing extraocular tissue and disinfected by rinsing 5 times with antibiotic water (10% penicillin-streptomycin in Phosphate-Buffered Saline (PBS) (−/−), Gibco, Paisly, UK). Removal of the anterior segment of the eye was obtained by cutting with sharp scissors at 5 mm distance from the limbus. After removal of the vitreous, the posterior eye cup was positioned in an individual well of a 6 well-plate. Next, eyecups were filled with heated (37 °C) EDTA-PBS (1 mM) solution and incubated at 37 °C in a humidified atmosphere containing 5% CO_2_ for 30 min. Afterwards, the retina was separated from the underlying RPE cells and choroid with a pincet and cut at the optic nerve. The eyecup was placed in an individual well of a new sterile 6-well plate, filled with heated (37 °C) 0.5% trypsin and incubated at 37 °C in a humidified atmosphere containing 5% CO_2_ for 30 min. Detachment of the pRPE cells was obtained by pipetting repeatedly trypsin solution in the eyecup and the final RPE cell suspension was transferred to a conical falcon tube. The cell suspension was diluted with culture medium (DMEM supplemented with 1% 2 mM l-glutamin, 2% penicillin/streptomycin and 10% FBS) and spun at 300× *g* for 5 min. Supernatant was discarded and the cell-pellet was resuspended in 10 mL pRPE cell culture medium (DMEM supplemented with 1% 2 mM L-glutamin, 1% penicillin/streptomycin, 10% FBS and 1% Non-Essential Amino Acids (NEAA)). The cell suspension was transferred to a T25 cell culture flask and incubated at 37 °C in a humidified atmosphere containing 5% CO_2_. Cell culture medium was refreshed three days post-harvesting. Three days before transfection, 6000 cells per well were seeded in 96-well plates.

The human Müller cell line, Moorfields/Institute of Ophtalmology-Müller 1 (MIO-M1) was obtained from the UCL Institute of Ophthalmology, London, UK. The cells were cultured in Dulbecco’s Modified Eagle’s Medium (DMEM) GlutaMax^TM^ pyruvate 1 g/L glucose (Gibco Invitrogen, Paisly, UK) supplemented with 1% 2 mM l-glutamin, 2% penicillin/streptomycin and 10% FBS (Hyclone^®^, Cramilton, UK). Cells were cultured in an incubator at 37 °C with humidified atmosphere containing 5% CO_2_ and passaged at 90% confluency. Five days before transfection, 2000 cells per well were seeded in 96-well plates.

In order to culture primary Müller cell glia, bovine eyes were obtained from the local slaughterhouse and transported in 4 °C CO_2_ independent medium (18045070, ThermoFisher^®^ Scientific, Merelbeke, Belgium). Eyes were cleaned by removing extraocular tissue and disinfected with antibiotic water (10% penicillin-streptomycin in PBS (−/−), Gibco, Paisly, UK). Removal of the anterior segment of the eye was obtained by cutting with sharp scissors at 5 mm distance from the limbus. After removal of the vitreous, the posterior eye cup was filled with CO_2_ independent medium. The eyecup was cut in 4 equal parts and the retina of 1 part was transferred to the gentleMACS^TM^ Octo Dissociator (Miltenyi Biotec, Bergisch Gladbach, Germany) containing separation medium. The latter consists of Advanced DMEM (Gibco^®^, Paisly, UK) supplemented with 1% GLutamax and 1% penicillin-streptomycin. After dissociation, the dissociated retina was transferred to a 40 µm filter (Corning Incorporated—Life Sciences, Durham, NC, USA) positioned on a 50 mL conical tube and spun down at 300× *g* for 5 min at room temperature. The supernatant of the falcon tube was discarded and the pellet was resuspended in 10 mL separation medium. This washing step was repeated three times and finally the cell pellet was resuspended in Müller growth medium (separation medium supplemented with 10% heat-inactivated FBS (Hyclone, Cramilton, UK) and 4 ng/mL epidermal growth factor (Sigma-Aldrich, Bornem, Belgium). The cell suspension was transferred to a CellBIND^®^ T75 flask (Sigma-Aldrich, Bornem, Belgium) and incubated at 37 °C in a humidified atmosphere containing 5% CO_2_. The cell culture medium was repeatedly refreshed after 1 week. When the cells were cultured for 3 weeks, they were passaged by 0.25% trypsin in T75 cell culture flasks. Five days before transfection, 2000 cells per well were seeded in Corning^®^ 96-well CellBIND^®^ microplates.

### 2.3. mRNA Transfection and B18R Treatment

The 60 kDa recombinant vaccinia virus protein B18R was purchased from ThermoFisher^®^ (ThermoFisher^®^ Scientific, Merelbeke, Belgium) dissolved in PBS with a concentration of 0.5 mg/mL. Cell medium was supplemented with B18R at a concentration of 150 ng/mL simultaneously with eGFP mRNA transfection. In order to evaluate the effect of B18R on eGFP mRNA expression, a suboptimal concentration of 0.05 µg/well mRNA was used. Therefore, mRNA was complexed with Lipofectamine^TM^ MessengerMAX^TM^ (ThermoFisher^®^ Scientific, Merelbeke, Belgium) at a cationic lipid-to-mRNA ratio of 3:1 (*v:w*). After transfection, cells were incubated at 37 °C in a humidified atmosphere containing 5% CO_2_ for 24 h. Fluorescence signal of Cy^®^5 and eGFP, for uptake and transfection experiments respectively, were measured by flow cytometry.

### 2.4. Flow Cytometry

After 24 h incubation with B18R and mRNA loaded lipoplexes, cell culture medium was removed and cells were washed once with 100 µL PBS (−/−) per well. Next, detachment of the cells was obtained by addition of 0.25% trypsin-EDTA (Gibco, Paisly, UK). After 5 min incubation at 37 °C, the trypsin was neutralized by adding 100 µL cell culture medium and the well plate was centrifuged at 500× *g* during 5 min. The supernatant was removed and cells were resuspended in flow buffer (PBS (−/−) supplemented with 0.1% sodium azide and 1% bovine serum albumin). Fluorescence signals were measured by using a CytoFlex (Beckman Coulter, Suarlée, Belgium) flow cytometer and data analysis was performed with FlowJo^TM^ software 10.5.3 (Treestar Inc., Ashland, OR, USA).

### 2.5. MTT Assay

The viability of all cell cultures was evaluated with the MTT-assay, 24 h post-transfection with mRNA and B18R. First, cell culture medium was removed and replaced by fresh culture medium including 5 mg/mL 3-(4,5-dimethyl-2-thiazolyl)-2,5-diphenyl-2*H*-tetrazolium bromide (MTT) reagent (Sigma-Aldrich, St. Louis, MO, USA) at a ratio of 1:9 (MTT reagent: culture medium). Next, the cells were incubated for 2 h at 37 °C in a humidified atmosphere containing 5% CO_2_. After incubation, the solution in the wells was removed and 100 µL DMSO (Sigma-Aldrich, St. Louis, MO, USA) was added to the cells to dissolve the formazan crystals. The well-plate was covered with aluminum foil and positioned on an orbital shaker (Rotamax 120, Heidolph, Germany) for 30 min at 1200 rpm. Lastly, the absorbance was measured at 590 nm and 690 nm (background) with a VICTOR3 1420-012 multilabel microplate reader (Perkin Elmer, Groningen, The Netherlands).

### 2.6. ELISA Assay

Supernatant of all cell types was collected 24 h after mRNA and B18R transfection and stored at −20 °C. The production of IFN-β and IFN-α was determined using the Human IFN-β ELISA kit (ThermoFisher^®^ Scientific, Merelbeke, Belgium) and the Human IFN-α kit (ThermoFisher^®^ Scientific, Merelbeke, Belgium) according to the manufacturer’s instructions.

### 2.7. Statistical Analysis

All data are the result of 3 independent experiments, unless declared otherwise, and are presented as mean ± standard deviation. Statistical analysis was performed with Graphpad Prism 8 software (San Diego, CA, USA). Analysis was performed by comparing the test samples with a single control group transfected without B18R addition, using a one-way ANOVA test followed by the Bonferroni post hoc test. Asterisks are used to illustrate statistical significance (* *p* < 0.05; ** *p* < 0.01; *** *p* < 0.001; **** *p* < 0.0001).

## 3. Results

### 3.1. Influence of B18R Treatment on Transfection Efficiency

The vaccinia virus-encoded protein B18R has often been used in cell reprogramming studies and has shown its capability to increase mRNA translation in different cell types [7,10,16,19,20,21]. Since mRNA is currently making progress as a potential therapeutic nucleic acid for retinal diseases, we explored the potential of B18R as an innate immune inhibitor in four ocular cell types: ARPE-19 cells, MIO-M1 cells, primary bovine RPE cells, and primary bovine Müller cells. B18R was added to the cell medium and cells were simultaneously transfected with mRNA-MessengerMAX lipoplexes containing a suboptimal mRNA concentration.

**Figure 1:**
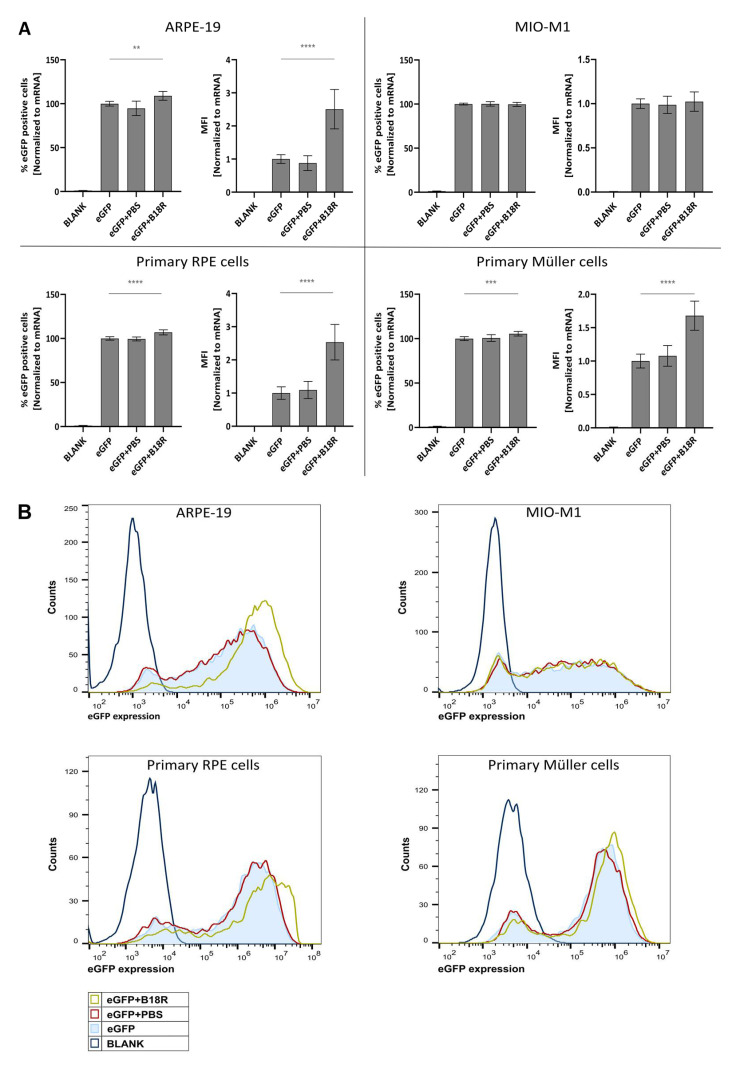
Transfection efficiency of mRNA-MessengerMAX lipoplexes when co-incubated with B18R. (**A**) Data are expressed as relative percentage enhanced Green Fluorescent Protein (eGFP) positive cells and relative Mean Fluorescence Intensity (MFI) both normalized to the average value of the control group which received only mRNA-MessengerMAX complexes (eGFP) (n = 3 × 3). Other samples represent untreated cells (Blank) or cells co-incubated with Phosphate-Buffered Saline (eGFP + PBS) or B18R (eGFP + B18R) during transfection. Mean absolute % of eGFP positive cells for the control group was 81%, 85%, 85% and 79% for the ARPE-19 cells, MIO-M1 cells, primary bovine RPE cells and primary bovine Müller cells respectively (** *p* < 0.01; *** *p* < 0.001; **** *p* < 0.0001). (**B**) Data represented as histograms obtained by flow cytometry.

As depicted in Figure 1, an increase in mRNA transfection efficiency can be observed for all cell types, except for the MIO-M1 cells. Moreover, an obvious resemblance between the ARPE-19 cells and primary bovine RPE cells can be noticed. Indeed, co-incubation with B18R increased the mean fluorescence intensity (MFI) in both cell types on average 2, 5 times. Statistical significant difference in % eGFP expression between cells treated with or without B18R was found, although a substantial increase cannot be noticed. Overall, we can conclude that for both types of RPE cells B18R is capable of increasing the degree of mRNA translation on average per cell without altering substantially the overall percentage of cells transfected.

In contrast to the RPE cells, the resemblance in transfection efficiency between MIO-M1 cells and primary bovine Müller cells was missing. Intriguingly, we did not see a difference in transfection efficiency between MIO-M1 cells transfected with or without B18R addition. However, in the primary Müller cells, B18R did enhance transfection efficiency, though less extensive than for the RPE cells. The MFI increased on average 1.7 times while the percentage of eGFP positive cells did not increase considerably, although it was considered statistically significant. At last, it must be noted that the addition of PBS, the B18R solvent, did not alter the MFI in any cell type which guarantees that the increase in MFI is exclusively due to B18R.

### 3.2. Influence of B18R Treatment on Uptake Efficiency

**Figure 2:**
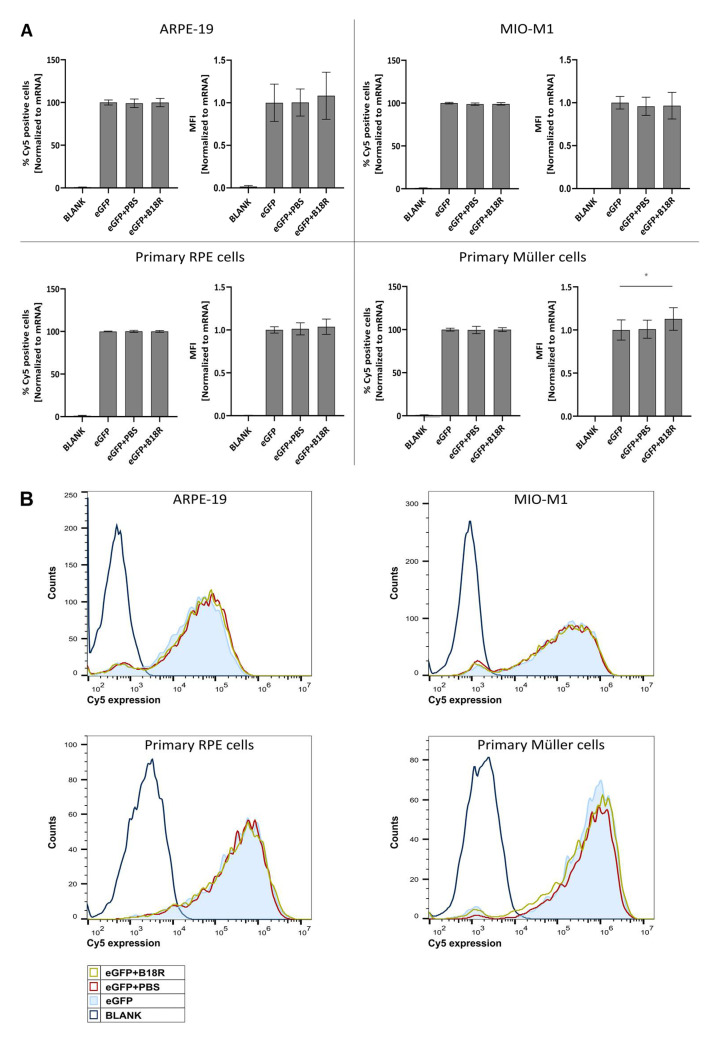
Uptake efficiency of mRNA-MessengerMAX complexes when co-incubated with B18R. (**A**) Data are expressed as the relative percentage of Cy^®^5 positive cells and relative MFI both normalized to the average value of the control group which received only mRNA-MessengerMAX complexes (eGFP) (n = 3 × 3). Other samples represent untreated cells (Blank) or cells co-incubated with PBS (eGFP + PBS) or B18R (eGFP + B18R) during transfection. Mean absolute percentage of Cy^®^5 positive cells was 84%, 96%, 95%, 98% for the ARPE-19 cells, MIO-M1 cells, primary bovine RPE cells and primary bovine Müller cells respectively (* *p* < 0.05). (**B**) Data represented as histograms obtained by flow cytometry.

Since we could observe a clear increase in effect for three out of four cell types, we wanted to investigate if the same trend was visible for uptake efficiency. Cy^®^5 labeled mRNA complexed with MessengerMAX was administered to the cells, whether or not in combination with B18R. 24 h after transfection the Cy^®^5 signal was determined by means of flow cytometry. Results depicted in Figure 2 show that the degree of uptake with or without B18R was rather the same. Except for the primary bovine Müller cells, a small but significant increase in the MFI was visible. Although when we compared this with the order of eGFP-MFI-increase it is clear that the latter could not be completely defined by an enhanced uptake. These data corroborate that rather than uptake, the effect of B18R is linked to intracellular processes. We hypothesize that the effect is mainly due to the decrease in innate immune system related type 1 IFNs concentration.

### 3.3. Influence of B18R Treatment on Type 1 IFN Production

Since B18R is classified as an innate immune inhibitor, we wanted to confirm if we could detect a decrease in type 1 IFNs secretion in the supernatant of the ocular cell types after transfection. All four cell types were transfected with mRNA-MessengerMAX lipoplexes alone or accompanied by simultaneous addition of B18R. 24 h after transfection, the supernatant of the cells was collected and preserved at −20 °C until the ELISA assay is carried out. We opted to determine the IFN-α and IFN-β concentration, as they are two classes of the type 1 IFNs and they are important factors in the innate immune reaction [7,10].

**Figure 3:**
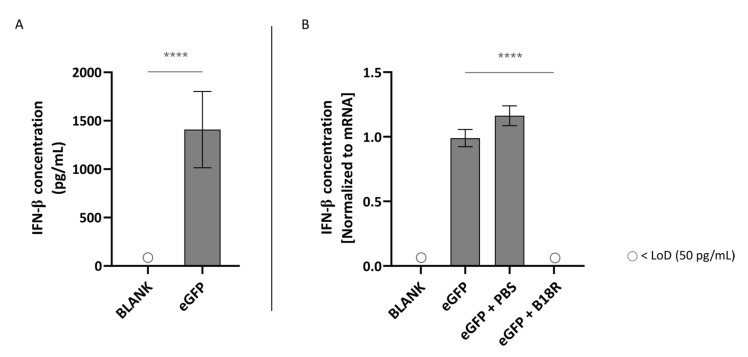
Interferon-β (IFN-β) production of ARPE-19 cells transfected with mRNA-MessengerMAX lipoplexes with or without B18R co-incubation as determined by ELISA (n = 3 × 3). (**A**) Absolute INF-β concentration in the supernatant 24 h following transfection of ARPE-19 cells with mRNA-MessengerMAX complexes without B18R addition. (**B**) IFN-β concentration, normalized to the group solely receiving mRNA-MessengerMAX complexes (eGFP), in the supernatant of ARPE-19 cells 24 h after transfection with mRNA-MessengerMAX complexes with or without B18R addition. The lowest concentration of IFN-β that could be detected with the used ELISA assay (LoD) was 50 pg/mL. Concentrations below the LoD are assigned with ○, **** *p* < 0.0001.

As depicted in Figure 3A, transfection of ARPE-19 cells with mRNA led to an extensive IFN-β production. More specifically, this resulted in a concentration of ~1409 pg/mL IFN-β in the supernatant of the cells. This confirmed that unmodified mRNA is indeed immunogenic and can hence efficiently trigger innate immune responses. However, possible partial immune stimulation by the carrier itself should also be taken into account. As displayed in Figure 3B, co-administration with B18R resulted in a spectacular drop in IFN-β concentration. In fact, the IFN-β concentration was lower than the limit of detection (LoD) of the ELISA assay. Since the control groups receiving PBS instead of B18R showed a small but significant increase in IFN-β concentration, we can guarantee that the decrease in IFN-β concentration is not owing to the B18R solvent.

**Figure 4:**
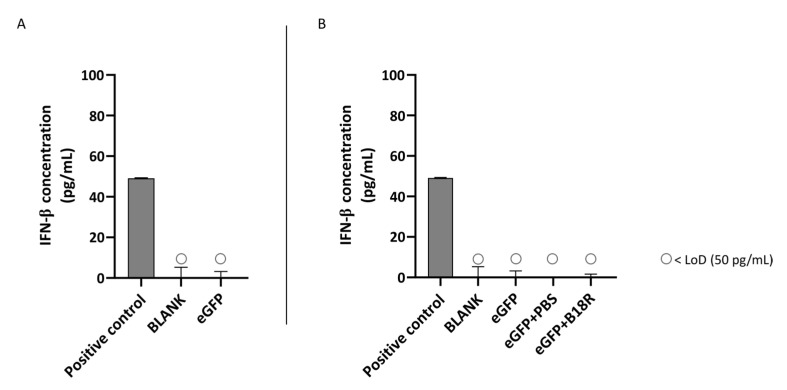
IFN-β production of MIO-M1 cells transfected with mRNA-MessengerMAX lipoplexes with or without B18R co-incubation as determined by ELISA (n = 3 × 3). (**A**) Absolute INF-β concentration in the supernatant 24 h following transfection of MIO-M1 cells with mRNA-MessengerMAX complexes without B18R addition. (**B**) IFN-β concentration, normalized to the group solely receiving mRNA-MessengerMAX complexes (eGFP), in the supernatant of MIO-M1 cells 24 h after transfection with mRNA-MessengerMAX complexes with or without B18R addition. The lowest concentration of IFN-β that could be detected with the used ELISA assay (LoD) was 50 pg/mL. Concentrations below the LoD are assigned with ○. Both graphs contain a positive control with a concentration of 50 pg/mL derived from the standard curve obtained during the experiment.

In contrast to the ARPE-19 cells, IFN-β production could not be induced by addition of mRNA-MessengerMAX lipoplexes to the MIO-M1 cells. Evidently, we could also not observe a decrease in IFN-β concentration after addition of B18R to the cells. All values of both control groups and test groups were lower than the LoD of the ELISA assay as depicted in Figure 4.

**Figure 5:**
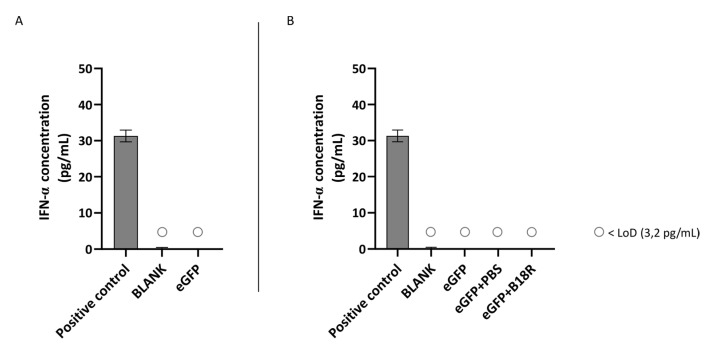
IFN-α production of ARPE-19 cells transfected with mRNA-MessengerMAX lipoplexes with or without B18R co-incubation as determined by ELISA (n = 3 × 3). (**A**) Absolute INF-α concentration in the supernatant 24 h following transfection of ARPE-19 cells with mRNA-MessengerMAX complexes without B18R addition. (**B**) IFN-α concentration, normalized to the group solely receiving mRNA-MessengerMAX complexes (eGFP), in the supernatant of ARPE-19 cells 24 h after transfection with mRNA-MessengerMAX complexes with or without B18R addition. The lowest concentration of IFN-α that could be detected with the used ELISA assay (LoD) was 3.2 pg/mL. Concentrations below the LoD are assigned with ○. Both graphs contain a positive control with a concentration of 31 pg/mL derived from the standard curve obtained during the experiment.

**Figure 6:**
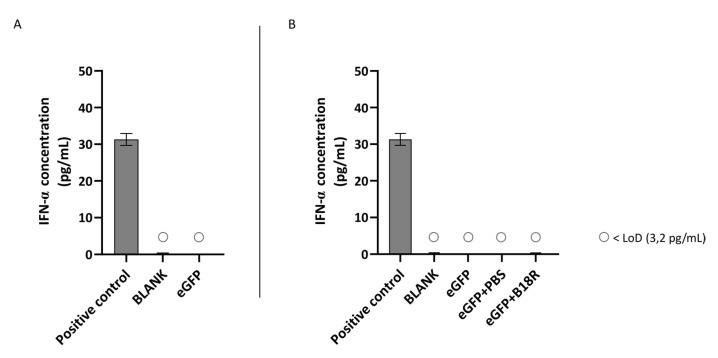
IFN-α production of MIO-M1 cells transfected with mRNA-MessengerMAX lipoplexes with or without B18R co-incubation as determined by ELISA (n = 3 × 3). (**A**) Absolute INF-α concentration in the supernatant 24 h following transfection of MIO-M1 cells with mRNA-MessengerMAX complexes without B18R addition. (**B**) IFN-α concentration, normalized to the group solely receiving mRNA-MessengerMAX complexes (eGFP), in the supernatant of MIO-M1 cells 24 h after transfection with mRNA-MessengerMAX complexes with or without B18R addition. The lowest concentration of IFN-α that could be detected with the used ELISA assay (LoD) was 3.2 pg/mL. Concentrations below the LoD are assigned with ○. Both graphs contain a positive control with a concentration of 31 pg/mL derived from the standard curve obtained during the experiment.

Another class of type 1 IFNs is IFN-α of which the concentration can also be determined by ELISA assay. An identical experimental set-up as for IFN-β was used. Results of these experiments are depicted in Figure 5 and Figure 6 for the ARPE-19 and MIO-M1 cells respectively. Unfortunately, for both the ARPE-19 cells and the MIO-M1 cells the IFN-α concentration in the cell medium was below the LoD of the ELISA assay. Consequently, a decrease in IFN-α concentration after B18R addition to the cells was impossible to observe.

### 3.4. Influence of B18R on Cell Viability

**Figure 7:**
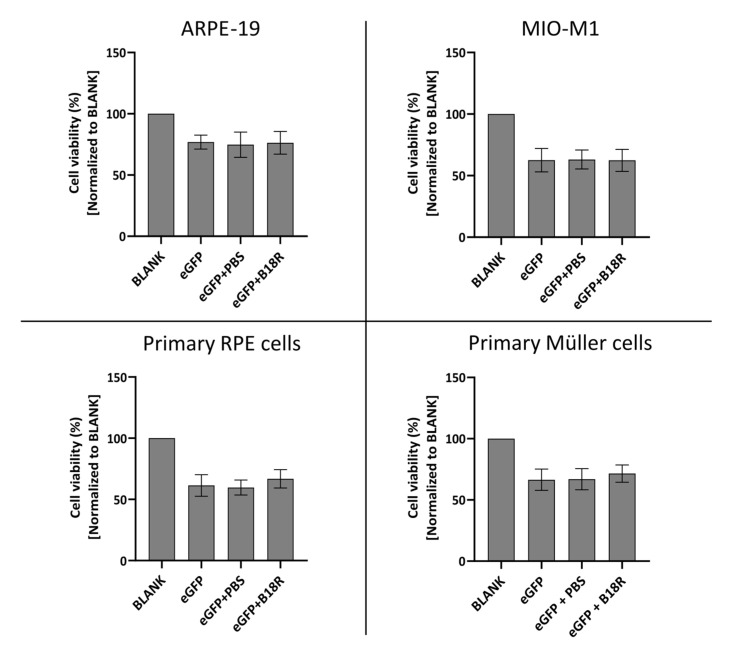
Cell viability following transfection with mRNA-MessengerMAX lipoplexes with or without co-treatment with B18R as determined by the MTT-assay. Read-out was performed 24 h after mRNA transfection. Data are expressed as the relative cell viability normalized to the untreated control group (BLANK) which did not receive any treatment. (n = 3 × 3).

Since B18R has shown to be a potential candidate for improving mRNA translation by reducing the innate immune system response after administration, it was interesting to evaluate its effect on cell viability. The degree of cytotoxicity was determined by MTT assay 24 h after incubation of the cells with MessengerMAX complexed mRNA, whether or not in combination with B18R. Results of these experiments are depicted in Figure 7 for all cell types.

Cell viability after supplementation of the cell medium with B18R is compared to the cell viability of the control group only receiving mRNA-MessengerMAX complexes. As depicted in Figure 7, a significant decrease in cell viability for cells treated with mRNA-MessengerMAX lipoplexes was observed when compared to the untreated cells. However, B18R did not lower the viability of any of the cell types studied in this research any further. Moreover, we did not notice substantial morphological changes between cells treated with or without B18R (data not shown). In contrast to what is reported in some studies, we did not observe an increase in cell viability upon B18R delivery to the cells. Finally, we could learn from this experiment that PBS did not induce any significant increase or decrease in cell viability. Again, this contributes to the conclusion that the effects we observed were solely due to B18R. Taken together we can conclude that no extensive cell death is observed after B18R treatment.

## 4. Discussion

**Figure 8:**
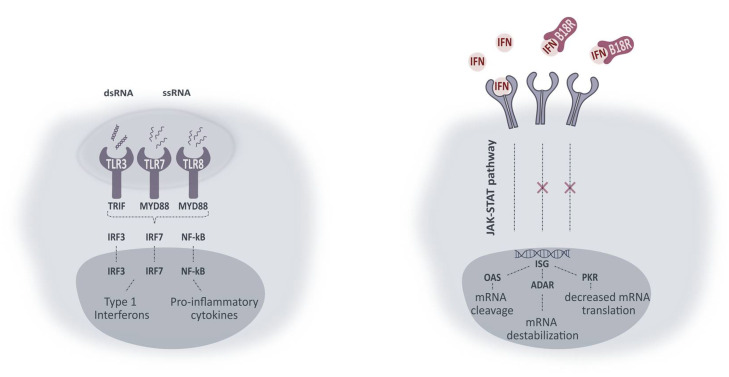
Simplified representation of intracellular pathways activated upon TLR activation and working mechanism of B18R. Activation of TLRs leads to interaction with adaptor molecules TRIF and MYD88. Initiation of successive pathways results in activated transcriptions factors (IRF3, IRF7 and NF-κB) causing production of type 1 IFNs and pro-inflammatory cytokines. Type 1 IFNs in their turn can activate IFN receptors in an autocrine or paracrine fashion. Activation of IFN receptors leads to the production of IFN-stimulated genes (ISG) via the JAK-STAT pathway. ISG can lead to production of proteins included in the signaling pathways (not shown) or proteins inducing mRNA damage or diminished mRNA translation. ssRNA, single-stranded RNA; ds RNA, double-stranded RNA; TLR, Toll-like receptor; TRIF, Toll-IL-1 receptor domain-containing adapter protein inducing IFN-β; MYD88, myeloid differentiation primary response protein 88; IRF, interferon regulatory factor; NF-kB, nuclear factor-κB; IFN, type 1 interferon; JAK-STAT, Janus kinase 1- Signal transducer activator of transcription; ISG, IFN-stimulated genes; OAS, 2’-5’-oligoadenylate synthetase; ADAR, RNA-specific adenosine deaminase; PKR, dsRNA dependent protein kinase.

mRNA made progress as a potential therapy for ocular genetic diseases but its profound immunogenicity still hampers the translation to the clinic. The induced innate immune response can result in decreased mRNA translation and/or increased mRNA degradation. Therefore, several strategies have been developed to block these undesirable effects. Some studies reported the effect of small molecule innate inhibitors on mRNA translation, yet with varying results [7,22,23,24,25,26,27]. One of these inhibitors is B18R which can be defined as a non-species-specific decoy receptor present in its soluble form in the cell medium or on the cell surface. Upon binding, type 1 IFNs are neutralized and both autocrine and paracrine transmembrane signaling is prevented [28,29,30]. A simplified schematic overview of intracellular pathways activated upon TLR-mRNA binding and working mechanism of B18R is presented in Figure 8. With the aim of improving mRNA translation efficiency, we wanted to evaluate the influence of B18R in different ocular cell types. In this regard, we delivered mRNA-MessengerMAX lipoplexes to ARPE-19 cells, MIO-M1 cells, primary bovine RPE and Müller cells with or without the addition of B18R to the cell culture medium. Transfection efficiency was always compared with cells only receiving mRNA-MessengerMAX lipoplexes. Furthermore, uptake efficiency, cell viability and type 1 IFN production were determined by flow cytometry, MTT assay and ELISA assay respectively.

As mentioned above, we made use of two immortal cell lines, ARPE-19 and MIO-M1, as a model for the human primary RPE and Müller cells [31,32,33]. These two ocular cell types are important in retinal homeostasis and encountered by medicines delivered via subretinal or intravitreal injection [11,13]. Due to several advantages, immortal cell lines became very attractive for investigation of biological processes and cellular responses to certain stimuli. Indeed, they are very cost-effective, easy to manipulate and can be cultured for an extended time. In addition, purchased cell lines are normally pure cell populations and therefore free of contamination [34]. However, it is worth mentioning that the latter seems to be not completely true as research has shown that cell type misidentification is highly present. A relevant drawback is that cells in culture can be subjected to mutations and chromosome alterations which can change their phenotype [35,36]. Taken together, commercial cell lines can be a convenient model for fundamental experiments but it can be useful to validate observations in primary cell types. For this reason, we opted to test the B18R treatment in two primary bovine cell types. Therefore, we could as well assess if interspecies differences are present.

First, we assessed the influence of B18R on transfection efficiency in the four ocular cell types described above. All cell types, except the MIO-M1 cells, showed an increased MFI after B18R treatment as depicted in Figure 1. Although B18R has a defined working mechanism and we are not the first research group to study its effect on mRNA translation, an increase in transfection efficiency is not always guaranteed [16,21,37]. Contradictory results have been described in several studies where B18R was delivered, mainly for reprogramming purposes. Yoshioka and co-workers transfected Human Foreskin Fibroblast (HFF) cells with a combination of GFP-encoding self-amplifying mRNA and B18R-mRNA. An increased GFP expression was noted when compared to the cells transfected with solely self-amplifying mRNA. Furthermore, due to B18R the duration of expression could be prolonged up to 7 days, necessary for effective reprogramming. Finally, they observed the same effect when the HFF cells were transfected with reprogramming factors-encoding self-amplifying mRNA and B18R-mRNA [20]. In contrast, Awe et al. did not notice an enhanced OCT4 expression when the encoding mRNA was delivered together with the B18R protein in human fibroblast cells. Even after using high concentrations of both B18R or mRNA, the increase in effect was missing [27]. Similarly, Drews et al. did not notice a decrease in innate immune system-related gene expression due to B18R or any of the other small molecules tested. Evidently, an upregulation in reprogramming gene expression was missing [28]. There are several studies describing the use of B18R alone or combined with other vaccinia virus proteins during transfection with reprogramming factor-encoding mRNA. However, no comparison is made in terms of transfection efficiency when B18R is not used. Lastly, in this regard Zangi and colleagues were the first research group to use B18R in vivo, injecting modified mRNA in a mouse myocardial infarction model [38]. As mentioned before, the ARPE-19 cell line showed high similarity with the primary bovine RPE cells in terms of transfection efficiency increase. To our knowledge, we are the first group to ever compare these two cell types in terms of B18R influence on mRNA transfection efficiency. Since both cell types are of different origin these results are quite interesting. Although immortalized and primary RPE cells showed high similarity, this was completely absent for the MIO-M1 cell line and the primary bovine Müller cells. This difference can be explained by several possible reasons which are described below.

Since B18R did not affect uptake efficiency of mRNA lipoplexes (Figure 2), the changes in transfection efficiency were highly likely due to intracellular processes and considering the clearly defined working mechanism of B18R we quantified the IFN-β concentration after B18R addition by means of an ELISA assay. Since ELISA assays are dependent on the origin of the cells used, we opted to test the two human cell lines for the IFN-β concentration present in the cell supernatant. To our knowledge, only Beissert et al. and Liu et al. showed the correlation between hampered IFN-β concentration and increased mRNA translation before [39,40]. Beissert and colleagues delivered the mRNA encoding for vaccinia virus proteins E3, K3 and B18R together with GFP-mRNA to HFF cells and showed that increased mRNA translation was clearly correlated with a tremendous decrease in IFN-β and OAS1 production. The enhanced expression of mRNA was also seen when the experiments were repeated in vivo [40]. The more recent study of Liu et al. validated the effect of B18R in HFF and HepG2 cells on mRNA transfection efficiency. However, they saw that NS1-TX91, a protein encoded by the eight mRNA segment of the influenza A virus, could induce a higher increase in effect and decrease in IFN-β production than B18R [39].

In this study, we showed that mRNA transfected ARPE-19 cells produce a high amount of IFN-β which is reduced to below the LoD after B18R administration. Therefore, we confirmed that the increase in transfection efficiency could be indeed directly correlated to a diminished IFN-β concentration. Considering the striking similarity in transfection efficiency between the human ARPE-19 and primary bovine RPE cells we suspect that a similar increase in IFN-β is present in the bovine RPE cells. Earlier studies described that the retinal pigment epithelium is a first line defense mechanism for pathogens, plays a pivotal role during infection to maintain the blood-retinal-barrier and can act as antigen presenting cells (APC). Furthermore, it has been shown that RPE cells play a role during infection and in the ocular innate immune reaction as they express several TLRs and can secrete several cytokines and type 1 IFNs in response to certain stimuli [41,42,43,44]. Kumar M. and co-workers were the first research group to define the expression of TLR1-7, 9 and 10 in human RPE cell cultures with real-time PCR. However, they only focused on the TLR3 signaling in detail since this TLR recognizes dsRNA which is an important feature of RNA viruses. When the human RPE cells were transfected with a dsRNA analog, they noticed that secretion of IFN-β but also Interleukin-6 (IL-6), IL-8, MCP-1 and sICAM-1 was induced. Due to the fact that they can act as APC and express TLR3, they show a lot of similarity with dendritic cells (DCs) although the latter also produce IFN-α upon activation [41]. However, the primarily ssRNA we delivered is not recognized by TLR3 but by TLR7/8 which triggers the MYD88 activator molecule transmitting the signal. This results in expression of several transcription factors which can initiate the production of type 1 IFNs and inflammatory cytokines [7,10]. Taken together, we can conclude that our results regarding IFN-β production are in line with previous reports in literature. Despite a clear presence of IFN-β observed after mRNA delivery to the ARPE-19 cells, they failed to produce IFN-α in reaction to the same stimulus. The lack of IFN-α production might not seem completely surprising as IFN-α is mainly excreted by plasmacytoid dendritic cells upon infection with mRNA or pDNA viruses [45]. However, several reports suggest that IFN-α might also be secreted by other cell types depending on the immune stimulus [46,47]. Kumar et al. previously showed that delivery of a dsRNA analog to human RPE cells induced the secretion of IFN-β but not of IFN-α. They attributed the lack of IFN-α production to the origin of RPE cells, the neural ectoderm, which is different from DCs derived from bone marrow and do express both IFN-β and IFN-α upon activation. They however only focused on TLR3 signaling since they found that TLR3 was one of the most highly expressed TLRs in RPE cells [41]. In light of our differing experimental conditions, i.e., primarily ssRNA and ordinarily activation of TLR7/8, production of IFN-α was not ruled out in our study. To elucidate which other possible cytokines are secreted and which TLRs are expressed to evaluate similarity with human RPE cell cultures further extensive analysis with immunohistochemistry, PCR, flow cytometry and/or Western blotting can be performed [48].

Although the ARPE-19 cells show a high production of IFN-β upon activation by mRNA, the opposite is true for the MIO-M1 cells. Here we could observe that there is no IFN-β production visible when mRNA is delivered to the cells. Hence, it was impossible to note a decrease in IFN-β concentration and therefore an effect of B18R. The reason why MIO-M1 cells did not produce IFN-β is not completely clear and somewhat surprising since it has been reported that Müller cells play an important role in innate immunity [42]. Kumar et al. was the first research group to elucidate the location and function of TLRs 1-10 in MIO-M1 glia, primary murine Müller cells and a mouse retina with RT-PCR. Furthermore, they demonstrated the presence of adaptor molecules MYD88, TRAM and TRIF which are important for activation of the signaling pathways. Finally, they detected an increased concentration of several cytokines and MAPKs of the NF-κB pathway after activation with TLR agonists. However, they did not evaluate type 1 IFN secretion or the presence of signaling molecules linked to other pathways. The group did show activation of TLR3, TLR7 and TLR9 after stimulation, showing that the Müller cells indeed have a role in innate immunity [48]. Additionally, other research groups described the production of several different cytokines by the MIO-M1 cell line and the gliotic retina [49,50,51]. Interestingly, we did see an increase in effect in the primary bovine Müller cells suggestive of IFN-β neutralization by B18R. This contrast with the MIO-M1 cell line can be explained by several factors. As described above, cell contamination might be a problem with immortal cell lines but can also happen while culturing the primary cells from a bovine retina. Although the purification protocol is partially selective and morphology of cells was evaluated before continuation of experiments, there is never 100% assurance that the final culture will only consist out of Müller cells. The contaminating cells, such as astrocytes, can possibly interfere with the results [33]. Another option is that there are intracellular differences between the MIO-M1 cells and primary bovine Müller glia. The latter are not only from another origin but might also be exposed to more stress than MIO-M1 cells, as described for rat primary Müller cells before [33]. This can possibly influence their reaction to components delivered to the cell. Similar to the ARPE-19 cells, no IFN-α production could be noticed after mRNA delivery to the MIO-M1 cells. Previous research showed that both IFN-α and IFN-β production could be stimulated in Müller cells both in vitro and in vivo after exposure to the herpes simplex virus type 1 [52]. However, the Herpes Simplex virus is a DNA virus and thus activates the immune system via the TLR9 and not via the TLR7/8 which may explain the difference in result [53,54]. Furthermore, we worked with a human immortal cell line and primary bovine Müller cells which are from a different origin than the Müller cells tested in the study of Drescher et al. These aspects might influence the outcome of experiments and therefore explain the difference of our study with results reported in literature regarding IFN-α production. However, since Kumar et al. showed presence of TLR7 in MIO-M1 cells, one would expect the production of type 1 IFNs after mRNA delivery [48]. We hypothesize that possibly one of the key players in the type 1 IFN-signaling cascade, such as IRF7, is not expressed. Further immunology research can be useful to detect if and which pathway and signaling molecules are activated upon mRNA delivery to MIO-M1 cells.

Finally, we evaluated the effect of B18R on the viability of the four different cell types. It has been reported before that B18R addition to the cell culture medium can improve cell viability after mRNA transfection [16]. We assume that this is also the reason several researchers use B18R in reprogramming studies with modified mRNA without comparison with the situation where no B18R is used [16,21,37,38]. Our results demonstrated no substantial changes in cell viability after treatment with B18R. Due to the difference with previously published results, we believe the reported increase in viability might be cell type-dependent. Furthermore, a more recent study showed that the increase in HFF cell viability due to B18R was only visible after multiple mRNA transfections [30]. This could also explain why we do not see a considerable increase in cell survival after one mRNA transfection.

## 5. Conclusions

In this study, we have proven that B18R can increase mRNA translation in ocular cell types. Since the increase in effect is mainly present in the RPE cells, both in the immortal cell line as the primary cells, the vaccinia virus protein can be primarily interesting as adjuvant during subretinal injections. Due to the presence of B18R, the concentration of the mRNA to be delivered can be downscaled while maintaining the same level of effect. This provides possibilities to reduce the degree of side effects that may occur. Although extensive immunology research was beyond the scope of this study, future tests on Müller cells to identify the signaling molecules activated and cytokines secreted upon mRNA delivery, can be interesting to elucidate the pathways that are induced and gain more knowledge about the intracellular processes that are involved. After all, the Müller cells remain an important cell type in the retina also reachable with the less invasive intravitreal injection technique. Additionally, possible added value of other vaccinia virus proteins could be evaluated and TLR expression analysis in primary bovine cells can be performed to elucidate resemblance with the immortal cell lines and estimate their potential as a good ex vivo model for retinal studies. At last, studying the effect of B18R on expression of mRNA encoding for a therapeutic protein of interest in the appropriate models would give us insight into its potential to become a clinically relevant therapy

## Data Availability

The data presented in this study are available in this article: Vaccinia Virus Protein B18R: Influence on mRNA Immunogenicity and Translation upon Non-Viral Delivery in Different Ocular Cell Types.
